# The effectiveness of a novel artificial intelligence (AI) model in detecting oral and dental diseases

**DOI:** 10.1038/s41405-025-00336-6

**Published:** 2025-06-30

**Authors:** Ravi Rathod, Saffa Dean, Christopher Sproat

**Affiliations:** 1 Guy’s and St Thomas’ NHS Foundation Trust, London, UK; 2 King’s College Hospital NHS Foundation Trust, London, UK

**Keywords:** Oral pathology, Preventive dentistry

## Abstract

**Introduction:**

Implementing artificial intelligence (AI) to use patient-provided intra-oral photos to detect possible pathologies represents a significant advancement in oral healthcare. AI algorithms can potentially use photographs to remotely detect issues, including caries, demineralisation, and mucosal abnormalities such as gingivitis.

**Aim:**

This study aims to assess the effectiveness of a newly developed AI model in detecting common oral pathologies from intra-oral images.

**Method:**

A unique AI machine-learning model was built using a convolutional neural network (CNN) model and trained using a dataset of over five thousand images. Ninety different unseen images were selected and presented to the AI model to test the accuracy of disease detection. The AI model’s performance was compared with answers provided by fifty-one dentists who reviewed the same ninety images. Both groups identified plaque, calculus, gingivitis, and caries in the images.

**Results:**

Among the 51 participating dentists, clinicians correctly diagnosed 82.09% of pathologies, while AI achieved 81.11%. Clinician diagnoses matched the AI’s results 81.02% of the time. Statistical analysis using *t*-tests at 95% and 99% confidence levels yielded p-values of 0.63 and 0.79 for different comparisons, with mean agreement rates of 81.55% and 95.11%, respectively. The findings support the hypothesis that the average AI answers are the same as average answers by dentists, as all p-values exceeded significance thresholds (*p* > 0.05).

**Conclusion:**

Despite current limitations, this study highlights the potential of machine learning AI models in the early detection and diagnosis of dental pathologies. AI integration has the scope to enhance clinicians’ diagnostic workflows in dentistry, with advancements in neural networks and machine learning poised to solidify its role as a valuable diagnostic aid.

## Introduction

Artificial Intellegence (AI) is advancing rapidly with multiple novel uses within all industries, particularly healthcare. A significant advancement has been the use of AI to address long patient waiting times for GP services and elective surgeries. By streamlining administrative processes, optimising appointment scheduling, and enhancing risk assessment, AI is improving accessibility and efficiency in healthcare, ultimately leading to better patient outcomes [1, 2].

A recent YouGov survey identified dental services as the most challenging National Health Service (NHS) to obtain. Prolonged waiting times are negatively impacting patient health, with one in five respondents reporting prolonged pain due to delayed treatment [3]. Limited access to dental care not only exacerbates discomfort but may also increase the risk of more severe oral conditions, highlighting the need for systemic improvements in diagnosis and accessibility.

Dentistry has recently seen a rise in the use of AI in different aspects of clinical dentistry, including pathological analysis [4], radiographic interpretation, and object detection.

AI is an adjunct that helps clinicians efficiently analyse and interpret data. The capacity in which it is being used is through image recognition [5–7] to localise objects/subjects on an image and undertake image classification similar to humans. Image recognition is advancing rapidly with multiple novel uses within all industries. This technology uses convolutional neural networks (CNNs) to be trained to recognise patterns that the human eye may not always see, with minimal or no human input. Advances in AI have exponentially increased the complexity of images that systems can process and reduced the human input required.

AI thrives when it has access to large quantities of data to learn from. Radiology and histopathology analysis and interpretation have seen great results with the use of algorithms to help diagnose pathologies from these standardised image forms [8–14]. AI removes the aspect of human error that can impact the analysis of pathology slides/radiographs when interpreting minute features [15, 16] and this attention to detail has allowed AI to show great promise in the accurate grading of oral squamous cell carcinoma (OSCC) [17, 18]

New and innovative ways to use AI are constantly arising. Studies in China found deep learning algorithms for detecting oral cancers as effective as experts and superior to medical students in identifying OSCC [19]. Additionally, Oka et al. demonstrated AI’s ability to recognise 23 dental instruments, achieving high image detection accuracy even with overlapping instruments [20].

This study aimed to evaluate the effectiveness of AI in detecting dental diseases from intra-oral photographs. We hypothesise that AI models can detect dental pathologies from intra-oral images at a level comparable to dental practitioners diagnosing the same images.

## Method

### Model development

For a CNN to learn to detect pathologies a large data set is required. Five thousand open-source images were collated from libraries such as Kaggle and Roboflow. Open-source data had a range of variants and was not biased towards one demographic. The patient’s medical history was not outlined or relevant to dental disease detection. Pathologies included were plaque, calculus, caries and gingivitis. Within each image were numerous diseases, complexities, and severity, which allowed us to obtain rich data to train our model accurately. To enable the model to distinguish between healthy and diseased tissues, the dataset included images without pathology.

Following the collection of the data set all images were labelled for plaque, calculus, caries and gingivitis. A team of 50 clinicians all with a minimum of 5 years of postgraduate experience were recruited to label the data set. Bounding boxes were manually drawn around all identified pathology within each image on all surfaces of teeth, irrespective of severity.

A team of employed machine learning engineers were recruited to develop a CNN using the labelled images. SSD was chosen as the preferred model for image detection in this scenario because of its ability to deliver fast results with high accuracy, as has been utilised in other studies of a similar nature [21–23].

The model was trained through 100,000 training loops to enable the algorithm to learn and demonstrate improvements across various metrics. The SSD model was evaluated against three different loss functions to optimise its performance for object detection:

1. **Classification loss:**
  This measures how well the model identifies areas of dental pathologies when compared with the actual areas labelled in the training data bounding boxes. It compares the predicted class probabilities with the actual labels to ensure accurate identification.
2. **Localisation Loss:**
  This measures the precision of the model in predicting the location of dental pathology. It compares the predicted bounding boxes with the ground truth and penalises differences to improve accuracy.
3. **Regularisation loss:**
  This prevents overfitting whereby the AI model ‘memorises’ unnecessary detail of the training data. Regularisation helps balance fitting the training data well and avoiding the memorisation of noise, leading to better performance on unseen data.
4. **Total loss:**
  The total loss combines all three losses. Minimising it ensures the model detects objects accurately, locates them precisely, and remains simple enough to work well on unseen data.

**Fig. 1:**
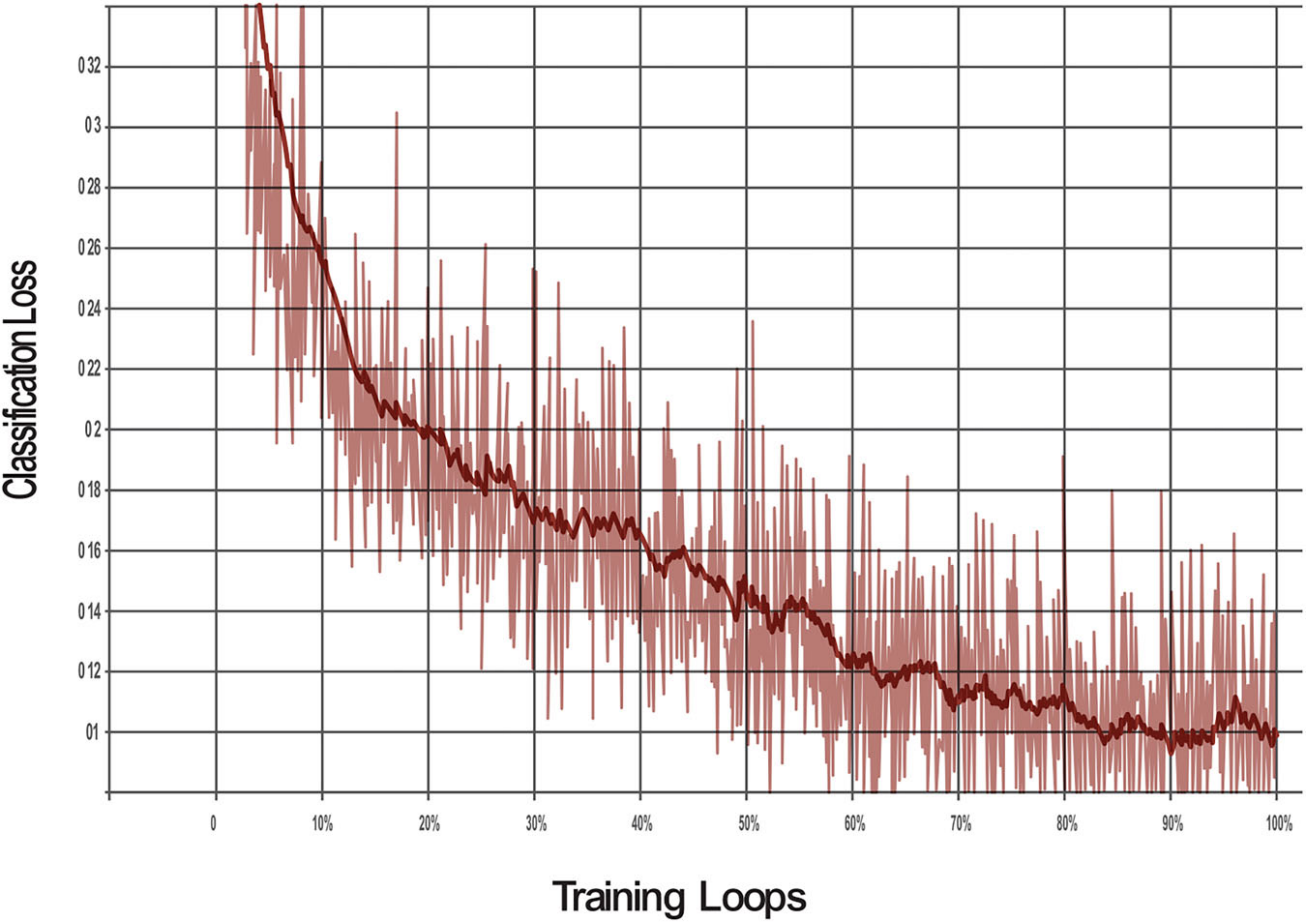
This graph shows the reduction in classifcation loss as the number of training loops increases.

**Fig. 2:**
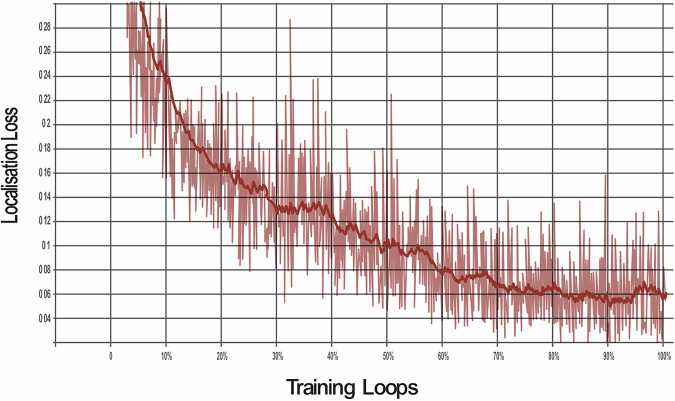
This graph shows the reduction in loss fucntion as the number of training loops increases.

**Fig. 3:**
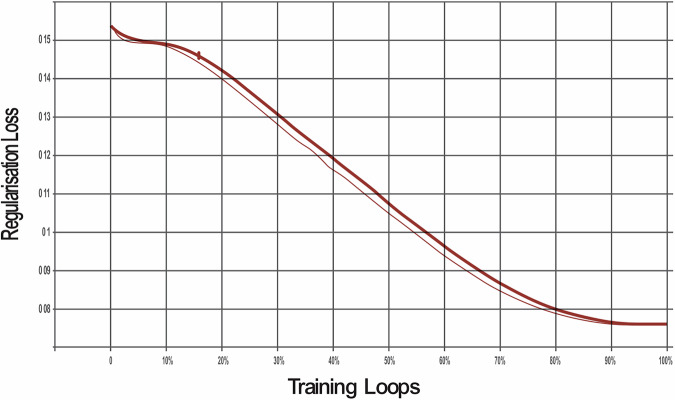
This graph shows the reduction in regularisation loss as the number of training loops increases.

**Fig. 4:**
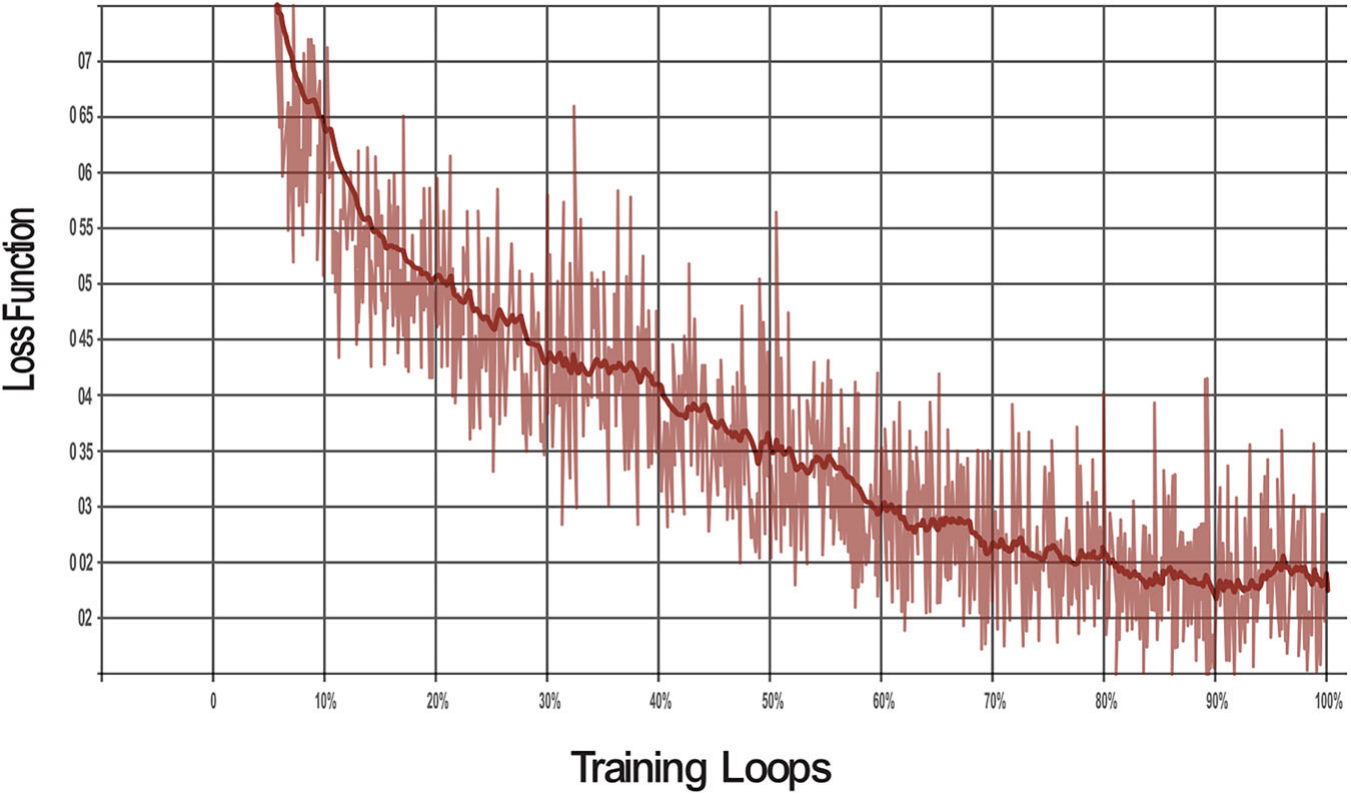
This graph shows the reduction in loss function as the number of training loops increases.

The classification loss was reduced by 91% (Fig. 1), the localisation loss was decreased to 92% (Fig. 2) and the regularisation loss was decreased by 95% (Fig. 3). The cumulative impact is reflected in the total loss, which was 78% (Fig. 4).

### Model testing

A panel of ten dentists with varying experience levels (general dental practitioners, hospital trainees and specialists) were recruited to independently annotate two hundred new, unseen open-source images for plaque, calculus, gingivitis and caries only. The dataset, similar to the images used to train the AI, included images of varying orientations, qualities and sizes. Only images for which all ten panel members provided identical annotations were included in the study, resulting in a final dataset of 90 new patient images, unseen by the AI model.

The AI model was tested with the new 90 unseen images and tasked with placing bounding boxes in areas it identified as dental pathology. For this study, pathology was only considered correct if the bounding box (the rectangle drawn around the object) was placed accurately. By ensuring precise placement, the study aimed to reduce false positives.

The performance of the AI model was tested against a control group of fifty-one (*N* = 51) clinicians, not involved with the study. The clinicians were of differing clinical backgrounds and experience levels, including core trainees, associates and dental specialists. Given that the study aimed to evaluate the AI model’s real-world applicability and clinical relevance, a representative sample of dentists with varying levels of expertise was deemed sufficient to provide meaningful comparisons without the need for formal power calculations. The 51 participants were asked to identify pathologies (plaque, calculus, caries and gingivitis only) on the same 90 images presented to the AI model via an online form. Responses from participants implied tacit consent to be used for analysis of performance. This study did not require ethical approval as no patients were directly involved and the study posed minimal risk and did not involve direct patient intervention.

## Results

**Fig. 5:**
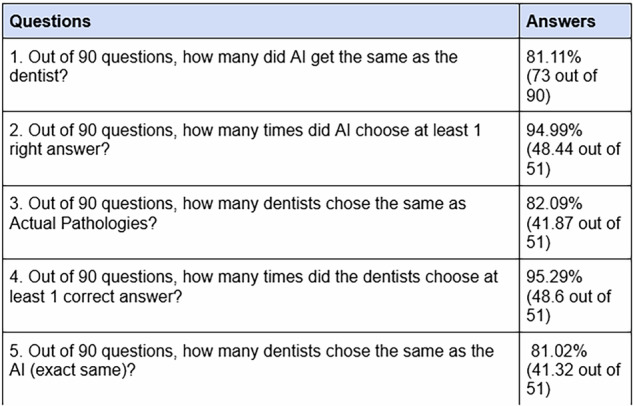
The table summarises the results from recipients compared to the AI model.

The results show that of the ninety patient images analysed, the AI model could identify at least one correct pathology in 94.99% of cases. These results are comparable to that of the 95.29% of dentists who could identify at least one correct pathology. Overall, 81.02% of dentists submitted the same responses as the AI. (Fig. 5).

**Fig. 6:**
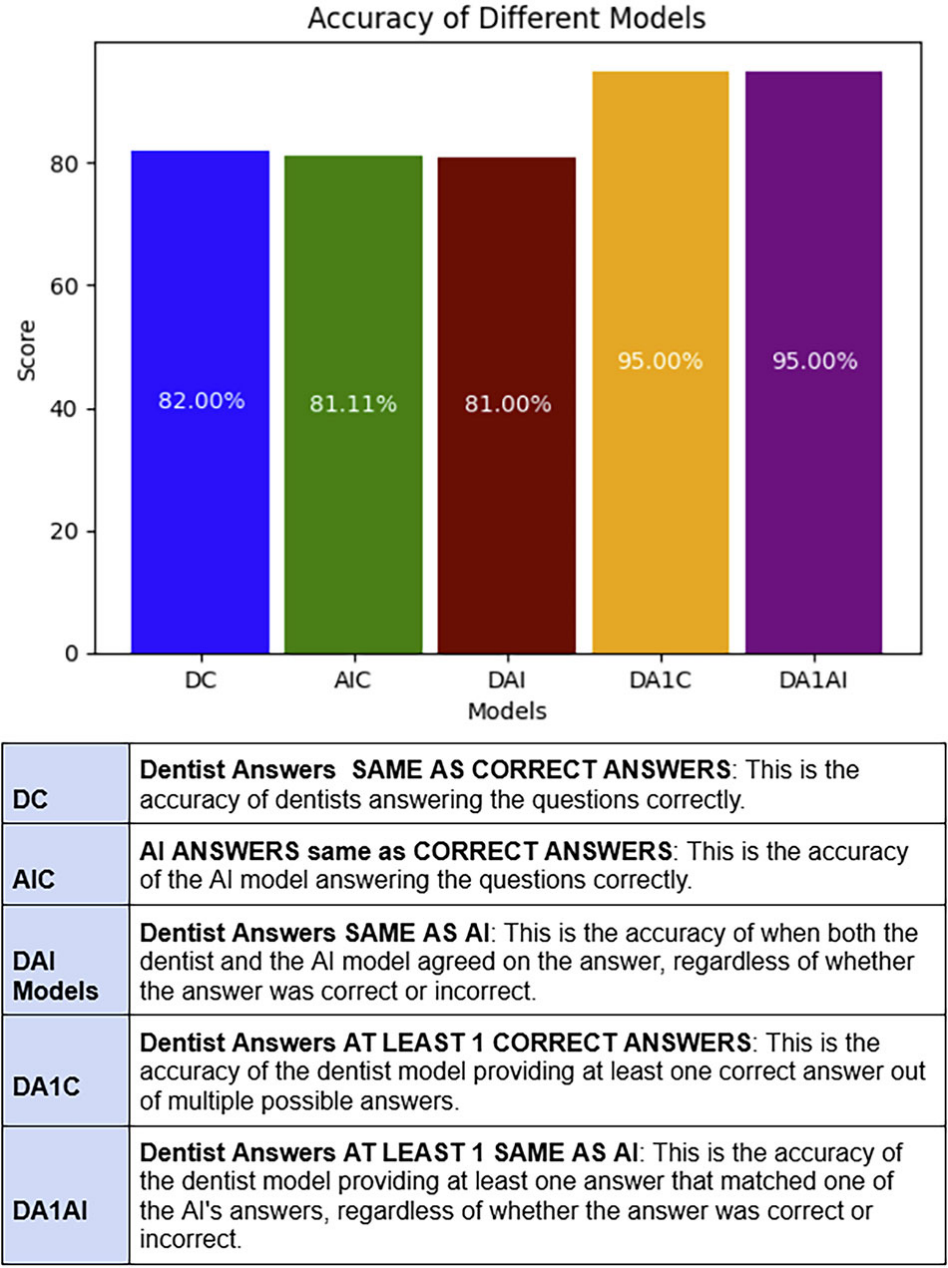
This graph shows the accuracy of different scenarios tested.

The graph below shows the percentage accuracy of different models analysed using the questionnaire results. The x-axis lists the five models that were evaluated: DC, AIC, DAI, DA1C, and DA1AI. The y-axis shows the accuracy as a percentage. (Fig. 6).

Overall, the graph shows that dentists achieved the highest accuracy in correctly answering the questions. However, the dentist model, which allowed for one or more answers (DA1C), and the dentist model, which allowed for one or more answers that matched the AI (DA1AI) had slightly higher accuracy than the model where the dentist had to match the same answer as the AI exactly(DAI). (Fig. 6).

**Fig. 7:**
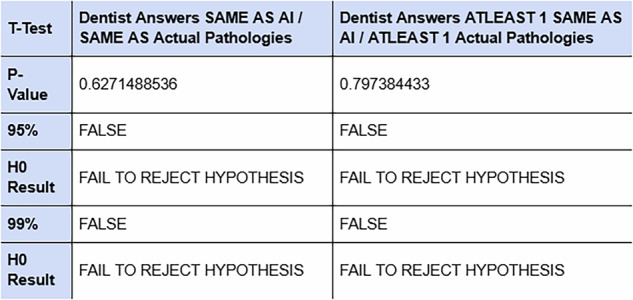
This table shows the results of the T-test performed.

After comparing the AI model’s results with clinicians’ answers, we determined whether there were any significant differences between the means of the two groups’ answers - a T-test was used to assess this. The image below (Fig. 7) summarises the hypotheses and the accuracy value obtained following testing.

The null hypothesis that AI answers differ from average dentists’ answers was shown to be false as AI and clinicians returned a high confidence value of *P* = 0.63 and *P* = 0.80, respectively (Fig. 7).

**Fig. 8:**
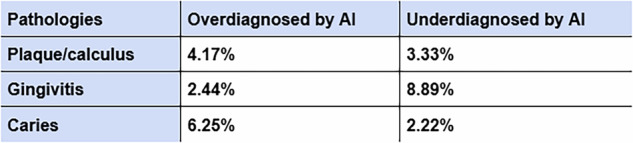
This table compares the instances where a pathology was incorrectly identified (overdiagnosed) and missed (underdiagnosed).

An in-depth review of the AI’s incorrect predictions indicated that missed diagnoses of gingivitis were relatively common (8.89%), whereas missed cases of caries were less frequent (2.22%). Conversely, the AI showed a propensity to overdiagnose caries (6.25%) and was least likely to incorrectly identify gingivitis when it was absent (2.44%) (Fig. 8).

## Discussion

Object detection models is a form of deep learning AI technology that can be employed in several sectors, especially in the healthcare sector. The ability to identify and classify pathology through images via teledentistry has the potential to significantly improve access and efficiency cost-effectively for a dental healthcare system that is currently overwhelmed. This study demonstrates the application of artificial intelligence (AI) in detecting oral pathologies from intraoral images and reimagining the delivery of patient care and triage pathways. The results provide supportive evidence for the efficiency, accuracy, and potential impact of AI in disease detection within dentistry.

Several AI state-of-the-art (SOTA) models are available for object detections including YOLO, Faster R-CNN, DETR and SSD. Studies comparing models evaluate accuracy alongside speed and computational power. The performance of SSD 300 ×300 is comparable to other SOTA models. Models such as YOLO and Faster R-CNN have been found to offer better accuracy and faster speed [24]. however, greater computational demands. The SSD 300 × 300 is recognised as a ‘lightweight’ model that requires less computing power [25]. This model was selected and developed with a view of its potential ease of implementation amongst an array of electronic devices including common mobiles and laptops for both patients and clinicians.

Our model is novel, as images are not always in the correct orientation or angle. The images used in this study were collated from various angles, lighting, and complexities, reflecting how we expect typical images to be sent remotely by patients. There is robust evidence suggesting that images in different resolutions, orientations, and formats can influence diagnostic results [26–28]. Over time, we can develop further models that would first orient the image to standardise detection, which will enhance the outcomes and confidence of disease detection by models like ours. Unlike AI in pathology or radiology, our model’s photos are directly captured from any device, in any orientation and camera quality, with varying colours, lighting, and sizes. The model can detect gingivitis, which appears as a red area of the gum, without confusing it with the labial fold, a deeper region by the lip that is also typically red due to its rich vascular supply. The same applies to its plaque-detection capability. Areas of tooth wear expose the yellow dentine, which may be mistaken for plaque. As the model advances, we can aim to achieve better differentiation to reduce limitations regarding angulation, lighting, or camera quality.

The developed model exhibited good accuracy, identifying 81.11% of pathologies correctly (Fig. 5). Although the number of diseases screened was limited to the major dental diseases in this current model, this result is encouraging, given the significant variability in the images used. Current challenges include training the model on a limited dataset of 5000 images - the performance is likely to improve as the model undergoes further training [29, 30]. The AI algorithm achieved an 81% similarity in pathologies detected compared to those identified by trained clinicians. When comparing the performance of both groups, only a 0.3% difference in the capability to identify at least one pathology correctly was observed - reinforcing the potential for AI as a screening aid.

Future evaluations of the AI model performance can be tested using key metrics like precision, recall, and F1-score. These metrics provide a measure of accuracy and reliability, and ensure the model is not simply memorising data. Validating the model on further unseen datasets, refinements can be made on its predictive capabilities and minimise errors. This iterative process is essential to optimising the AI system for real-world clinical applications in dental practice [31, 31].

Further analysis of the algorithm’s incorrect responses indicated that gingivitis was the most frequently missed pathology. A possible explanation for this is the wide colour variation in the presentation of gingivitis, further impacted by image quality and lighting conditions. Many images we have trained the data on were captured using mobile phone cameras. Additional complications, such as pigmentation or confusion with labial folds, or prominences around canines, where the bone density is greater, can cause some optical ambiguities. Conversely, caries was the most frequently overdiagnosed and the least often missed pathology. This suggests that while the current version of the algorithm shows high sensitivity for caries, its accuracy remains lower than for other pathologies. The presence of black triangles, stained fissures, and shadows on teeth may contribute to these over-diagnoses. This is a prime example of where clinicians excel, as they can correlate findings to their location within the oral cavity to facilitate accurate diagnosis.

## Conclusion

The study highlights that the current AI model developed is comparable to dental clinicians in diagnosing intra-oral pathologies. Further iterations of the model will be assessed with the aim to improve the diagnostic efficiency and accuracy and broadening the range of pathologies detected. With improved diagnostic power, further studies will explore the impact of AI in helping to reduce clinicians’ workload and the potential application for improving patient access.

## Data Availability

All data used in this study were obtained from open-source materials. The datasets analysed during the current study are available from the corresponding author upon reasonable request.
